# Supplementary material for: A Novel Drosophila Model of Alzheimer’s Disease to Study Aβ Proteotoxicity in the Digestive Tract
Source: Int J Mol Sci. 2024 Feb 9;25(4):2105. doi: 10.3390/ijms25042105 (PMC10888607; doi:10.3390/ijms25042105)
Supplement: Supplementary file 1 [file ijms-25-02105-s001.zip › ijms-2783778-supplementary.pdf]

Supplementary figures

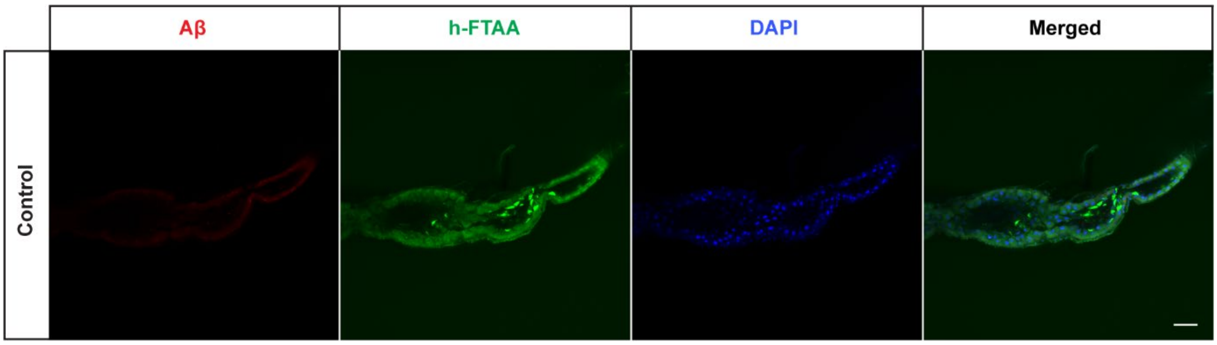

Figure S1: Detection of h-FTAA fluorescence in the midgut of Drosophila control flies using the Myo31DF driver. *Confocal microscope single-plane images showing the midgut of control flies stained with Mabtech anti-human Aβ antibody (red) and LCO ligand h-FTAA (green). The sections have been counterstained with DAPI (blue) to visualize cell nuclei. Scalebar, 50 µm.*

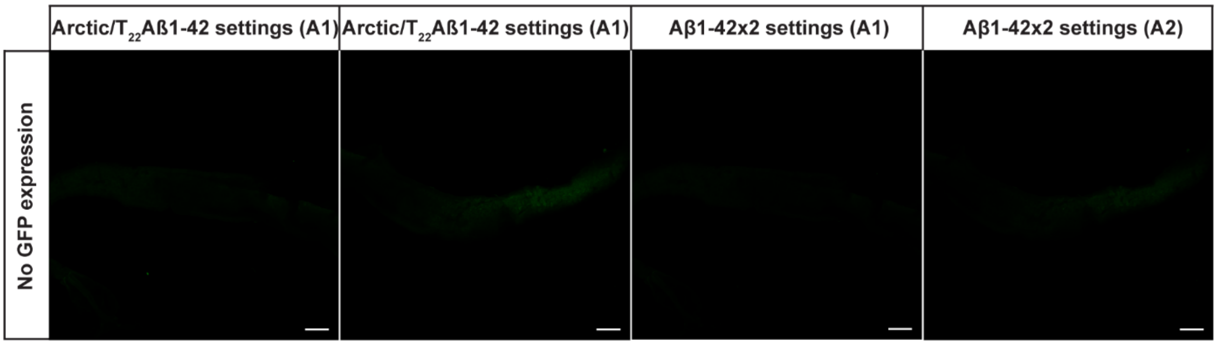

Figure S2: *Detection of GFP expression baseline using flies without the driver gene Myo31DF and the apoptotic sensor UAS-GC3Ai. Scalebar, 100 µm.*
